# A Conceptual Muddle: An Empirical Analysis of the Use of ‘Sex’ and ‘Gender’ in ‘Gender-Specific Medicine’ Journals

**DOI:** 10.1371/journal.pone.0034193

**Published:** 2012-04-18

**Authors:** Anne Hammarström, Ellen Annandale

**Affiliations:** 1 Department of Public Health and Clinical Medicine, Umeå Centre for Gender Studies in Medicine, Research Project Challenging Gender, Umeå University, Umeå, Sweden; 2 Department of Sociology, University of Leicester, Leicester, United Kingdom; 3 Umeå Centre for Gender Studies in Medicine, Research Project Challenging Gender, Umeå University, Umeå, Sweden; University of British Columbia, Canada

## Abstract

**Background:**

At the same time as there is increasing awareness in medicine of the risks of exaggerating differences between men and women, there is a growing professional movement of ‘gender-specific medicine’ which is directed towards analysing ‘sex’ and ‘gender’ differences. The aim of this article is to empirically explore how the concepts of ‘sex’ and ‘gender’ are used in the new field of ‘gender-specific medicine’, as reflected in two medical journals which are foundational to this relatively new field.

**Method and Principal Findings:**

The data consist of all articles from the first issue of each journal in 2004 and an issue published three years later (n = 43). In addition, all editorials over this period were included (n = 61). Quantitative and qualitative content analyses were undertaken by the authors.

Less than half of the 104 papers used the concepts of ‘sex’ and ‘gender’. Less than 1 in 10 papers attempted any definition of the concepts. Overall, the given definitions were simple, unspecific and created dualisms between men and women. Almost all papers which used the two concepts did so interchangeably, with any possible interplay between ‘sex’ and gender’ referred to only in six of the papers.

**Conclusion:**

The use of the concepts of ‘sex’ and gender’ in ‘gender-specific medicine’ is conceptually muddled. The simple, dualistic and individualised use of these concepts increases the risk of essentialism and reductivist thinking. It therefore highlights the need to clarify the use of the terms ‘sex’ and ‘gender’ in medical research and to develop more effective ways of conceptualising the interplay between ‘sex’ and ‘gender’ in relation to different diseases.

## Introduction

The processes of equating women with their biology and taking the male body as the norm or reference point have a long history within medicine [1]. The introduction of the concept of ‘gender’ into feminist research in the 1970s was an important counter to the prevalent perception of women's bodies as inferior to the bodies of men [1]. Whereas the term ‘sex’ generally is taken to refer to reproductive biological differences between men and women, the concept of ‘gender’ was introduced to distinguish biological ‘sex’ from the social, cultural, and historical construction of gender [2]. Thus, feminist research draws attention to the fact that differences between men and women are not constant or impervious to change [3], [4]. Rather, gender means how being a man and a woman are interpreted in different cultures and how masculinities and femininities are shaped continuously and differently across time and space [2]. These processes of gender construction are related to power differentials between men and women as well as to other asymmetrical power relations, such as those that may be based on age, social class, ethnicity and sexual orientation [5], [6]. ‘Gender’ thus concerns the social and cultural relationships through which sexed bodies and reproductive processes are incorporated into the social world [2].

Most medical researchers today acknowledge that both social/cultural and biological factors are important for men's and women's health. For example, women are diagnosed with depression twice as often as men in most Western countries [7] and major reviews conclude that this cannot be explained by biological factors alone [8], [9]. The interplay between ‘sex’ and ‘gender’ (i.e. how ‘sex’ and ‘gender’ are interrelated to or interact with each other) is vital to an understanding of the differential development of diseases in men and women. For example, Ann Fausto-Sterling has shown how our skeletons are part of the life process. Sexed biological bodily processes interact with surrounding gendered social and cultural events from birth throughout our lives which results in women being diagnosed more often than men with osteoporosis [10].

The concepts of ‘sex’ and ‘gender’ are therefore essential to an accurate analysis of many dimensions of health and illness of men and women. Yet, a highly cited publication from the U.S National Institute of Medicine (IOM) from the *Committee on Understanding the Biology of Sex and Gender Differences*, concluded that the concepts have been used in an inconsistent and confusing way in the scientific literature [11]. The quest for conceptual clarity in the use of ‘sex’ and ‘gender’ has been encouraged by organisations such as WHO [12], Health Canada [13], [14] and by many feminist researchers [15]–[17]. The IOM report provided inspiration for the growing professional movement of ‘gender-specific medicine’, which is defined by one of its major protagonists as “the science of how men and women differ in their normal function and in the experience of disease” (page 61) [18]. The movement of ‘gender-specific medicine’ is seemingly inspired by earlier feminist critique [3], [4], [19]–[21] of the prevailing male norm in medicine, i.e. that men are the standard even in studies of diseases that affect both men and women. The male norm often meant that women were excluded from trials and thus research findings were underreported for women [19]. The growing women's health movement contributed to political decisions in the early 1990s to demand the inclusion of women in research and therefore, gender blind research is less common today. [4], [19]. Yet at the same time the increasing amount of research including both men and women has brought new problems in its wake, so much so that, today, feminist researchers point to the problematic tendency to exaggerate biological differences between men and women [19], [22]. There is also growing awareness of the risks of such exaggeration beyond feminism. For example, a re-analysis of highly prominent claims for sex differences in genetics concluded that most could not be verified [23]. Several of the re-analysed articles used the concept of ‘gender-specific’ in their titles.

Given these concerns, an important question that remains to be answered is whether ‘gender-specific medicine’ has brought more clarity to the concepts of ‘sex’ and ‘gender’?

The aim of this article is to empirically explore how the concepts of ‘sex’ and ‘gender’ are used in the new field of ‘gender-specific medicine’, as reflected in two new medical journals which are foundational to this relatively new field.

## Materials and Methods

Two journals on ‘gender-specific medicine’, indexed in *Pub Med*, were launched in 2004. *Gender Medicine* (formerly *Journal of Women's Health and Gender-Based Medicine*) (GM) is the official journal of the Partnership for Gender-Specific Medicine at Columbia University, USA. Its self-defined aim is to focus “exclusively on the impact of ‘sex’ and ‘gender’ on normal human physiology and on the pathophysiology of disease” (page 1) [24]. The *Journal of Men's Health & Gender* (JMHG, renamed *Journal of Men's Health* from Vol 5, 2008) is the official journal of the International Society of Men's Health whose current president is also based at Columbia. As defined in its initial aims and scope, JMHG aims to “inform, educate, encourage debate and engender innovation in treatment and preventative medical care within the field of men's health and gender-specific medicine” (these Aims and Scope appeared without page number in the first Issue of JMHG).

### Material

The first issue of a journal is important since therein editorial boards draw attention to the journal's remit and direction through specially selected papers and endorsements. Thus, we chose all texts (i.e. all research articles, reviews/meta-analysis and editorials/commentaries) from the first issue of each first volume (May 2004, JMHG; August 2004, GM) for analysis. We also chose to analyse an issue three years after the launch issues to gain insight into the state-of-the-art of the field (March 2007, JMHG; June 2007, GM). In addition, it is useful to analyse the editorials as they often provide a wider lens into the remit and the content of the issues. Therefore, we selected every editorial and commentary from each issue of both journals covering the whole period of roughly 3 years (2004–2007). In total, 104 papers were included (see Table 1).

**Table 1:** Description of the material as well as the content (more than one content coded per paper) of the papers in the two journals.

	Gender Medicinen (% of total)	Journal of Men's Health and Gender, n (% of total)
Total articles assessed	36	68
Research articles, 2004	7 (19%)	16 (24%)
Research articles, 2007	7 (19%)	13 (19%)
Editorials/commentaries	22 (61%)	39 (57%)
*Content of research articles*		
Clinical differences between men and women	11 (31%)	7 (9%)
Treatment of specific disorders	8 (22%)	2 (3%)
Prognosis, risk factors	2 (6%)	3 (4%)
Attitudes, behaviour.	1 (3%)	1 (1%)
Utilization of health services	0	1 (1%)
Diagnosis/treatment of men	0	12 (18%)
Experimental studies on male-female differences	1 (3%)	1 (1%)
Epidemiological studies on male-female differences in health or health behaviours	2 (6%)	4 (6%)
Men's health (other topics)*	0	5 (7%)
*Content of editorials/commentaries*		
Support for gender-specific medicine	16 (44%)	8 (12%)
Critique of men's health movement	3 (8%)	0
Male disadvantages in treatment or research	0	19 (28%)
Drug treatments for women	3 (8%)	0
Drug treatments for men	0	9 (13%)
Policy issues	0	3 (4%)
Others	4 (11%)	18 (26%)**

* masculine identity, domestic violence.

** e.g research method, writing style for the journal, tobacco control, medical education, ethics.

As can be seen in Table 1, the content differed between the journals. While GM had a strong clinical focus on differences between men and women, JMHG focused more specifically on men's health. Many of the editorials in JMHG introduced the articles contained in the issue. While the GM editorials strongly argued for gender-specific medicine, an additional feature of JMHG was men's disadvantage as compared to women.

### Analysis

Quantitative (Table 2) and qualitative (Tables 3 and 4) content analyses were undertaken by the authors with a combination of inductive and deductive approaches [25].

**Table 2:** Quantitative content analyses of the papers from the two journals.

	*Gender Medicine* *n = 36*	*J. Men's Health & Gender n = 68*	Total n = 104
‘Sex’ is used	24 (67%)	29 (43%)	53 (51%)
‘Gender’ is used	22 (61%)	43 (63%)	65 (63%)
‘Sex’ is defined	2 (6%)	3 (4%)	5 (5%)
‘Gender’ is defined	2 (6%)	4 (6%)	6 (6%)
Both ‘sex’ and ‘gender’ are used	20 (56%)	24 (35%)	44 (42%)
‘Sex’ and ‘gender’ are usedinterchangeably	19 (53%)	20 (29%)	39 (38%)
Possible interplay between ‘sex’ and ‘gender’	2 (6%)	4 (6%)	6 (6%)

**Table 3:** Excerpted definitions, subcategories and categories in the qualitative coding of the concept ‘sex’*.

Excerpted definitions	Refers to the Committee [11] which “defined sex as the classification of living things as male or female based on their reproductive organs and functions” (page 13) [28]	“Sex” which refers to the biological characteristics that define humans as female or male” (page 7) [48]	“an understanding, beyond simple reproductive differences, of the complex biological factors that affect the health of men and women” (page 19) [29]
	Sex (nature, e.g. genes and hormones) (page 13) [28]	Sex (biological) (page 20) [30]	
	“Sex reflecting a male or a female individual based on chromosomal complement and physical characteristics” (page 6) [49]	Sex (being male or female) (page 17) [28]	
Subcategories	Reproductive origins, dualism	Nonspecific biological differences	Beyond simple reproductive differences
Categories	Simple biological differences		More complex biological differences

* based on the 5 papers that defined ‘sex’.

**Table 4:** Excerpted definitions, subcategories and categories in the qualitative coding of the concept ‘gender’*.

Excerpted definitions	“Gender being an individual's self-representation, shaped by biology as well as responses to environment, experiences and societal factors” (page 7) [49]	“Gender refers to the array of …. personality traits, attitudes, behaviours, values, … that society ascribes to the two sexes on a differential basis” (page 7) [48]	“Gender refers to the array of socially constructed roles and relationships, …….., relative power and influence that society ascribes to the two sexes on a differential basis” (page 7) [48]	“Gender or socially structured factors” (page 19) [29]	Gender as ‘socio-cultural aspects of health’ (page 20) [30]
	“gender, a uniquely human concept, as a person's self-representation as male or female, which is rooted in biology and shared by environment and experience” (page 13) [28]		“gender (nurture, environmental factors and experience)” (page 13) [28]	“Gender is used here to refer to the social construction of roles, responsibilities, opportunities, and expectations related to being either male or female.” (page 21) [29]	
Subcategories	Self-representation and Dualism	Personality traits, Attitudes, Behaviours and Dualism	Relationships, Power, Environment and Dualism	Social constructions and Dualism	Socio-cultural aspects of health
Categories	Dualistic individualised focus		Dualistic societal approach		Gender and health

* based on the 6 papers that defined ‘gender’.

All 104 papers were carefully read with the intention of grasping the content of the text. Then an *inductive qualitative content analysis* was performed in the following way [26], [27]. All text that contained definitions of the concepts of ‘sex’ and ‘gender’ was excerpted and read through several times [27]. Thereafter, the text was sorted and abstracted into preliminary subcategories, which in turn were sorted and abstracted into preliminary categories [27]. A category answers the question ‘What?’ and refers mainly to a descriptive level of content. A category often includes a number of sub-categories at varying levels of abstraction. The preliminary subcategories and categories were discussed, reflected on and condensed into final subcategories and categories. The excerpted definitions, subcategories and categories are presented in Tables 3 and 4. For example, one paper [28] provided the following three different excerpted definitions of ‘sex’:

1. ‘Sex’ as the classification of living things as male or female based on their reproductive organs and functions” (page 13)
2. ‘Sex’ (nature, e.g. genes and hormones) (page 13)
3. ‘Sex’ (being male or female) (page 17)

The first definition is centred on how reproductive organs and functions differ between men and women and was coded and abstracted into the two subcategories ‘reproductive origin’ and ‘dualism’ (i.e. a focus on differences between men and women). While both the second and the third definitions were brief, the second (with focus on genes and hormones) could also be coded as ‘reproductive origin’, while the third was coded as ‘nonspecific biological differences’. The three subcategories (‘reproductive origin’, ‘dualism’ and ‘nonspecific biological differences’) were abstracted into the category: ‘simple’.

Also, all text related to an interplay between ‘sex’ and ‘gender’ (i.e. text about how ‘sex’ and ‘gender’ were interrelated or interacted with each other) was excerpted and shown in the Results section. Also, the number of papers which made reference to any interplay, was counted.

Reading and analysing the text raised the following new research questions:

Are the concepts of ‘sex’ and/or ‘gender’ used in the papers?
Are the concepts of ‘sex’ and ‘gender’ defined?
Are the concepts of ‘sex’ and ‘gender’ used interchangeably i.e. do the authors give them the same meaning?

The articles were re-read and coded in order to answer these new questions. A final *quantitative content analysis* was then undertaken [26] in which the number of articles for the new research questions was counted (see Table 2). Also, the number of papers which referred to any interplay between ‘sex’ and ‘gender’ was counted.

Methodological triangulation was performed in different ways in order to increase the trustworthiness of the data and its interpretation [26]. First, a combination of qualitative and quantitative methods was used. Second, from this, the two investigators, from two different fields (sociology, EA and medicine/public health, AH), coded the definitions of ‘sex’ and ‘gender’ independently and thereafter the codes and subcategories were compared. Only minor disagreements were found and, where that occurred, the coding was discussed until agreement was reached.

## Results

The quantitative content analysis (Table 2) shows that the concept of ‘sex’ was used in 53 of 104 papers, while ‘gender’ was used somewhat more often in 65 of the 104 papers. Both ‘sex’ and ‘gender’ were used in 44 of the 104 papers. However, these concepts were seldom defined: ‘sex’ was defined in five while ‘gender’ was defined in six of the papers. In fact (as can be seen from Tables 3 and 4), it was the same five papers in which ‘sex’ and ‘gender’ were defined while ‘gender’ was solely defined in additionally one paper. In 39 of the papers, the concepts were used interchangeably, which means that both ‘sex’ and ‘gender’ were used in relation to, for example, biological differences between men and women. The papers used the concepts interchangeably, irrespective of whether they were defined or not.

### Definitions

Interestingly, no definitions of the concepts of ‘sex’ and ‘gender’ were provided in any of the editorials in GM, while they are defined three times (twice in the first Issue) in editorials of JMHG.

The definitions of ‘sex’ and ‘gender’ in the papers are excerpted in Tables 3 and 4.

Table 3 shows that two categories – ‘simple biological differences’ and ‘more complex biological differences’ were identified in the five papers. Most often a simple definition of ‘sex’ was provided, referring to subcategories such as reproductive origins, dualism, nonspecific biological differences and ‘beyond simple reproductive differences’. Such papers give simple and limited definitions of ‘sex’, often only in a few words in the paper as a whole, and do not go on to integrate the definition into the analyses which follow. Only one paper gives a more complex definition with strong arguments of the need for more complex analyses of biological differences between men and women beyond simple reproductive differences e.g. visceral sensitivity and central nervous system pain processing. [29]. It is notable that there were no papers addressing health policy issues (such as inequities in health) in GM.

Table 4 shows that three categories were identified in the definitions of ‘gender’ (in five different papers). The first category ‘dualistic individualised focus’ was derived from several identified subcategories (self-representation, dualism, personality traits, attitudes and behaviours). The second category ‘dualistic societal approach’ was derived from the subcategories of relationships, power, environment, dualism and social constructions. The third category ‘gender and health’ was constructed from the subcategory socio-cultural aspects of health.

Overall the definitions of ‘sex’ and ‘gender’ contained a strong focus on dualisms i.e. differences between men and women, typically leaving variations among men and among women not conceptualised. Mostly ‘sex’ and ‘gender’ were implicitly treated as constant and fixed (rather than as variable and modifiable).

### Interplay between ‘sex’ and ‘gender’

Only six out of the 44 articles which used both ‘sex’ and ‘gender’ across both journals made direct reference to any possible interaction or interplay between ‘sex’ and ‘gender’. The interplay in these six papers was referred to in the following ways. An editorial in JMHG (with reference to the IOM Committee [11]) emphasised the importance of “the critical interlinked interactions of nature (genes and sex hormones) and nurture (environment and experience) on behaviour and perception” (page 12) [28]. Another editorial in the same issue stated that, “it seems highly likely that biological, social, psychological and behavioural variables interact to produce many important gender differences” (page 21) [30]. An article about the state of men's health in Europe concluded that men are at a higher risk of developing nearly all of the major diseases which affect both men and women and that in order to understand this, “it is necessary to look both at the biological entity of man and the role men's perception of their masculinity has in their attitude towards their health” (page 64). [31]. In a JMHG paper about sexual function Alessandra Graziottin strongly argues for a better understanding of the biological sexual similarities between men and women and ‘their dialectic and continuous relation with biological and socio-culturally related sexual differences’ (page 77) [32]. She emphasises the importance of neuroplasticity and psychoplasticity as ‘basic mechanisms that bridge together and re-shape the individual biological and psychological world through the continuous interaction with the environment’ (page 77).

Sarah Payne's paper [29] is the only one amongst the six that systematically analyses how ‘sex’ and ‘gender’ interact. Also (as can be seen from Tables 3 and 4) her paper can be categorised both as approaching gender as a socially structured factor and as providing a more complex understanding of ‘sex’ that goes beyond simple reproductive differences. Payne's paper concluded that it is difficult to disentangle ‘sex’ and ‘gender’ in the aetiology of irritable bowel syndrome and therefore that a complex model of the interaction between ‘sex’ and ‘gender’-linked factors is needed [29]. In an editorial in GM, Legato also attempted to focus on this interplay as she referred to Nobel prize winner Eric Kandel, quoting him as follows: “the structure of the brain is not fixed, but that *experience actually modifies its anatomy and neurochemistry*” (page 60, emphasis in original) [33]. Legato concluded that ‘brain sex’ may not be as fixed as we think and that, “perhaps the most difficult issue is to decide what about our brains is different because of the sex-specific interplay between our genes and hormones and the impact of our experiences on brain structure and function” (page 60) [33].

Thus, in summary, our results showed that less than half of the 104 papers used both concepts of ‘sex’ and ‘gender’. Less than one in ten papers attempted any definition of the concepts. Overall, the given definitions were simple, unspecific and created dualisms between men and women. Almost all papers which used the two concepts did so interchangeably. Any possible interplay between ‘sex’ and ‘gender’ was referred to only in six of the papers.

## Discussion

Our analysis points to the need for greater clarity in the use of the concepts of ‘sex’ and ‘gender’, as already proposed by the IOM report [11]. Interestingly, while much of the content of this report forms the basis for the movement of ‘gender-specific medicine’, our analysis shows that the papers published in these two journals championing this new field have failed to heed one of its guiding precepts. Even the editorials make no declaration of the need for conceptual clarity. The IOM report refers to analyses [34] of the use of ‘sex’ and ‘gender’ in journals indexed in *Pubmed* which found that more than half of the articles did not distinguish between the terms. Our analysis shows that 39 out of 44 articles in the two journals endorsing ‘gender-specific medicine’ used the concepts interchangeably, suggesting that the situation has deteriorated rather than improved with time.

Our analyses showed that ‘gender’ typically was used in a dichotomous way in the two journals, neglecting the feminist research on the importance of analysing multiple forms of gender constructions [2], [5], [6]. Related to this, the analysed papers did not question whether ‘sex’ should always be regarded as dichotomous or if ‘sex’ could be viewed as a continuum with multiple variations on the X and Y chromosomes. Such approaches to medical research and practice could be important, despite the additional complexity involved. As Hanson relates, ‘the imposition of a dichotomy on a continuum is…like dividing mercury with a ruler’. As she continues, much like mercury, the phenomena reconstitute themselves into a whole when the ruler is taken away (page 57) [35].

The inappropriate separation highlighted by Hanson is shared by critics of the growing emphasis on categorical difference in both ‘gender-specific medicine’ and more widely in medical science and healthcare [36]. The dualisms created in the papers of ‘gender-specific medicine’ in relation to the use of ‘sex’ and ‘gender’ increase the risk of exaggerating both biological and socio-cultural differences between men and women. Exaggerations are of course very problematic and not in line with demands on scientific rigour. Also, dualism increases the risk of biological essentialism, i.e. the tendency to regard sex and gender-related differences as unchangeable and as valid for *all* men and women, irrespectively of culture, time, and place [19]. This tendency is at odds with the recent emphasis on intersectionality in feminist research [1], [2], [6] which focuses on how various social power relations (of gender, socioeconomic position, ethnicity etc) are interrelated to each other.

As shown in earlier discourse analysis of the papers included in this study, much of ‘gender-specific medicine’ is directed towards high technology hospital medicine [37], such as ‘gender-specific pharmacology’. Such an approach pays little, if any, attention to the social dimensions of disease.. This could explain why few of the analysed papers included any discussion of a possible interplay between ‘sex’ and ‘gender’ in spite of the growing awareness within biomedicine more widely of the environmental impact on diseases. For example, the growing interest in epigenetics [38] focuses on the interplay between biological factors and the socio-cultural environment.

Launching GM, the Editor-in-Chief focused mainly on the impact of ‘sex’ in medicine in relation to the definition of ‘gender-specific medicine’ (“the science of how men and women differ in their normal human function and the experience of disease”) (page 61) [18]. Also, the journal has ‘gender’ rather than ‘sex’ in the title and the GM editorials provide no clear reason for this. The statement that the journal focuses “exclusively on the impact of ‘sex’ and ‘gender’ on normal human physiology and on the pathophysiology of disease” (page 1) [24] once again shows, with no rationale for why both terms are being used, the intention to use ‘gender’ in relation to biochemical processes rather than in relation to the socio-cultural context.

Thus, the dominating biomedical discourse in ‘gender-specific medicine’ (especially in GM) [37] contributes to making the socio-cultural context invisible. In the words of the founding editor-in-chief of GM, ‘gender-specific medicine’ marks a shift away from ‘old school’ views of women's health as ‘a feminist…issue’ towards a science of biological sex differences) [39]. Thus Legato seems to regard socio-cultural influences on women's health as limited. Yet as Hanson states, ‘biomedical research is using an inappropriate separation that may have more social than physical relevance’ (page 57) [35]. A main message in the conception of ‘gender-specific medicine’ is the location of health and especially illness in individual bodies rather than in the wider social arrangements of society. This is at odds with recent research which highlights the importance of the development of the physical body in interaction with a changing social world for medical practice [10], [40].

GM does not make reference to the feminist theoretical literature. Although neither JMHG attends to feminist literature and debate, comparatively speaking it pays greater consideration to social ‘gender’ and also makes some reference to the women's health movement and men's health movement. As seen in Table 1 (and as we have discussed elsewhere [37]), many of the authors argue that there is a men's health crisis, citing examples in men's social disadvantage in treatment or research vis a vis women.

In contrast to GM, the concepts of ‘sex’ and ‘gender’ are mentioned and defined in some of the editorials in JMHG. ‘Gender- medicine’ appears in the aims and scope and there is reference to the definitions of ‘sex’ and ‘gender’ from the IOM report [11]. But the term ‘gender’ was dropped from the title from the start of 2008. The given reason was to avoid the association between ‘gender’ and women i.e. to direct attention to *men*. Nonetheless, the Editor-in-Chief claims that “the discussion of gender – as other social, behavioural, attitudinal, cultural, and clinical phenomena - remains squarely within JMH's editorial purview”(page 3) [41]. Rather ironically, GM retains ‘gender’ in its title, but directs its attention principally to biology, while JMH has dropped ‘gender’ yet claims to preserve a focus on social factors or the gendering of men's health.

An important question that remains to be answered is *why* the concepts of ‘sex’ and ‘gender’ are so profoundly muddled in two journals which have as their aim the development of research related to ‘gender-specific medicine’, a movement which calls for more research about ‘sex’ and ‘gender’ in medicine. One explanation could be the contradictions which lie in the IOM report itself. Biology is defined as including the social environment: “the genetic, molecular, biochemical, hormonal, cellular, physiological, behavioural and psychosocial aspects of life” (page 13) [11]. Such a definition of biology would imply that the concept of ‘sex’ also includes social aspects. But the report [11] defined ‘sex’ only in biological terms. Thus, the IOM report urges clarity regarding the concepts of ‘sex’ and ‘gender’ but generates a confused mix of the concepts itself.

Another possible explanation for the conceptual confusion is that ‘gender-specific medicine’ in itself is so vague a concept that a claim for clarity of its basic concepts would be damaging - since, in the case of most papers, ‘gender’ is actually reduced to ‘sex’ (and/or the social reduced to the biological) [37]. As we have argued elsewhere [37], this reductivist thinking is consistent with the approach to research and clinical practice that underpins much of ‘gender-specific medicine’ which emphasises high technology, hospital-based medicine. ‘Gender-specific medicine’ is also a signal opportunity to extend pharmaceutical markets in the search for ‘gender-specific’ treatments [37]. It is notable that the Partnership for Gender-Specific Medicine in the USA lists several pharmaceutical companies including Procter and Gamble, Wyeth, Bayer Corporation, and Pfizer as supporters [42] and a board member is listed as advisor and member of the steering committees for various pharmaceutical companies, including Pfizer, Astra Zeneca, and Eli Lilly. [43].

Finally, what does the word “*specific*” imply? Throughout the editorials, this is never explained. Our analyses show that it might mean a focus on differences; that is, on the biological or socio-cultural specificity of men and women. When the concept of ‘gender’ was introduced into research on health in the 1970s it was explicitly intended to signal that differences between men and women are as much social as biological. Differences are consequently variable, being influenced by the changing social contexts of men's and women's lives [1]. Drawing attention to this variability would be inconsistent with the emphasis of gender-specific medicine on essential differences and also appears to be counter to its message and interests. Moreover the emphasis on gender *specific* is reductivist. That is, typically ‘gender’ is reduced to a property of individuals, evident in the focus on social roles (as properties of individuals) and individual perceptions favoured by many of the authors. In the texts reviewed, these are only loosely, if at all, connected to wider social structures such as societal gender ideologies, the ‘gendering’ of the economy, and socio-economic patterns of dis/advantage. These points are reinforced in Table 1 where we demonstrate that only few papers discuss policy issues such as health disparities.

As several authors have argued, bodies have agency in relation to their environment as they constantly respond to both inside and outside changes [10], [29], [44]. Although they are integral to this interactive process, our understanding of the exact role that ‘sex’ and ‘gender’ play is limited to date. As Krieger explains, ‘although lucid analyses have been written on why it is important to distinguish between ‘gender’ and ‘sex’, epidemiological and other health research has been hampered by a lack of clear conceptual models for considering *both*, simultaneously, to determine their relevance - or not – to the outcome(s) being researched’ (page 653) [45]. Following our analysis of papers in GM and JMHG, our worry is that ‘gender-specific’ medicine will actively inhibit the development of the clear conceptual models that researchers like Krieger quite appropriately call for. It signals a return to biological determinism [45], to dualistic conceptualisations of sex/biology and social/gender, and reveals the emptying out of ‘gender’ as a meaningful category as it is reduced to individual ‘lifestyle’ characteristics or social roles.

A possible clinical implication of our findings is that the confused way of handling the concepts of ‘sex’ and ‘gender’ in ‘gender-specific medicine’ could lead to an inappropriate understanding of the aetiology behind self-reported illness, medically diagnosed disease, and sickness (i.e. social aspects of disease, e.g. sickness absence from paid work) in men and women, which in its turn could lead to inappropriate diagnosis and treatment. One example of this from our analyses is an article about the prognostic role of ‘gender’ in lung-cancer survival [46]. The authors use the terms ‘sex’ and ‘gender’ interchangeably without defining them. They conclude (on page 45) that “female gender exerted a significant positive effect on survival after lung resection for patients with stage I”. Thus, when considering the aetiology behind their findings, they use the concept of ‘gender’ (which should be related to social and cultural factors), when talking about biomedical factors (reproductive hormones, receptors). Only one socio-cultural factor (smoking) is mentioned. This is an example of how authors use ‘gender’ when they appear to mean ‘sex’. If the reader took the term ‘gender’ at face value (i.e. did not realise that the authors were using it when they really mean sex/biology), then their attention would be directed towards finding socio-cultural factors to explain women's longer survival when, in this case, they should be concerned with biological explanations. However, as smoking, of course, also has significant physiological/biological effects, this is also an example of the importance of visualizing the interplay between ‘sex’ and ‘gender’ in order to understand diseases, including lung cancer.

One of the papers could serve as a good example of how to undertake an integrated analysis of the interplay between ‘sex’ and ‘gender’ [29]. The paper drew on two concepts from Krieger & Zierler [47] to illustrate the synergy between ‘sex’ and ‘gender’ in relation to Irritable Bowel Syndrome (IBS). “Gendered expression of biology” illustrates how biology can influence ‘gender’; biological differences between men and women that affect IBS-related pain are interpreted by both the woman and the medical profession through a gender lens, which influenced both individual experiences/expressions of the condition and the consultation and treatment [29]. The concept “biological expression of ‘gender’” referred to “the ways in which gendered differences may be incorporated and expressed by the body, and become physical or biological” (page 25). For example, it was concluded (page 25) that IBS is related to sexual abuse and a possible explanation could be that “physical alterations in the hypothalamic-pituitary adrenal axis in response to childhood sexual abuse can be seen as both biological and as the result of the gender relations in which such abuse occurs.” Most researchers today agree on the need to analyse the role of both socio-cultural and biological factors in the aetiology of illness, disease and sickness [8]–[10].

### Conclusion

The use of the concepts of ‘sex’ and gender’ in ‘gender-specific medicine’ is conceptually muddled. The simple, dualistic and individualised use of these concepts increases the risk of essentialism and reductivist thinking, It therefore highlights the need to clarify the use of the terms ‘sex’ and ‘gender’ in medical research and to develop more effective ways of conceptualising the interplay between ‘sex’ and ‘gender’ in relation to different diseases.
